# Cerebral Perfusion Effects of Cognitive Training and Transcranial Direct Current Stimulation in Mild-Moderate TBI

**DOI:** 10.3389/fneur.2020.545174

**Published:** 2020-10-07

**Authors:** Davin K. Quinn, Joel Upston, Thomas Jones, Emma Brandt, Jacqueline Story-Remer, Violet Fratzke, J. Kevin Wilson, Rebecca Rieger, Michael A. Hunter, Darbi Gill, Jessica D. Richardson, Richard Campbell, Vincent P. Clark, Ronald A. Yeo, Claude William Shuttleworth, Andrew R. Mayer

**Affiliations:** ^1^Department of Psychiatry and Behavioral Sciences, University of New Mexico, Albuquerque, NM, United States; ^2^Department of Neuroscience, University of New Mexico, Albuquerque, NM, United States; ^3^Chicago Medical School, Chicago, IL, United States; ^4^Naval Health Research Center, San Diego, CA, United States; ^5^Department of Speech and Hearing Sciences, University of New Mexico, Albuquerque, NM, United States; ^6^Department of Psychology, University of New Mexico, Albuquerque, NM, United States; ^7^Mind Research Network, Albuquerque, NM, United States

**Keywords:** traumatic brain injury, cognitive training, cerebral perfusion, pCASL, transcranial direct current stimulation

## Abstract

**Background:** Persistent post-traumatic symptoms (PPS) after traumatic brain injury (TBI) can lead to significant chronic functional impairment. Pseudocontinuous arterial spin labeling (pCASL) has been used in multiple studies to explore changes in cerebral blood flow (CBF) that may result in acute and chronic TBI, and is a promising neuroimaging modality for assessing response to therapies.

**Methods:** Twenty-four subjects with chronic mild-moderate TBI (mmTBI) were enrolled in a pilot study of 10 days of computerized executive function training combined with active or sham anodal transcranial direct current stimulation (tDCS) for treatment of cognitive PPS. Behavioral surveys, neuropsychological testing, and magnetic resonance imaging (MRI) with pCASL sequences to assess global and regional CBF were obtained before and after the training protocol.

**Results:** Robust improvements in depression, anxiety, complex attention, and executive function were seen in both active and sham groups between the baseline and post-treatment visits. Global CBF decreased over time, with differences in regional CBF noted in the right inferior frontal gyrus (IFG). Active stimulation was associated with static or increased CBF in the right IFG, whereas sham was associated with reduced CBF. Neuropsychological performance and behavioral symptoms were not associated with changes in CBF.

**Discussion:** The current study suggests a complex picture between mmTBI, cerebral perfusion, and recovery. Changes in CBF may result from physiologic effect of the intervention, compensatory neural mechanisms, or confounding factors. Limitations include a small sample size and heterogenous injury sample, but these findings suggest promising directions for future studies of cognitive training paradigms in mmTBI.

## Background

A significant minority of patients with mild traumatic brain injury (mTBI), up to 33%, go on to experience functional impairment a year later (1, 2). These persistent post-traumatic symptoms (PPS) can range from the somatic (dizziness, headaches, light sensitivity) to the cognitive (difficulty focusing, impaired memory) and emotional realms (depression, irritability, anxiety) (3). Historically these were thought to be due to poor coping with stress, or malingering (4, 5). However, advanced imaging has contributed significantly to our current understanding of the acute and chronic sequelae of mTBI, and expanded the possible etiologies of PPS to not only include psychological phenomena but also neurological factors (6–10).

Candidate mechanisms of PPS include microscopic axonal shearing and microhemorrhage (11); functional connectivity abnormalities (7); and ongoing neuroinflammation (12). One of the more promising hypotheses receiving significant scientific attention is that of abnormal cerebral perfusion, in which traumatic injury causes impaired neurovascular coupling and mismatch between neuronal metabolic demand and cerebral blood flow (CBF) (13–16). There is ample grounding for this pathophysiology in animal models and in severe TBI in humans (17–19), however, noninvasively detecting changes in CBF in more mild injuries has proved more challenging (20). Arterial spin-labeling (ASL) and pseudocontinuous ASL (pCASL) magnetic resonance imaging sequences are techniques for measuring cerebral perfusion that have gained traction recently. It has permitted quantification of both global and regional CBF without use of injected or inhaled agents (21, 22), based on the premise of magnetically labeling arterial blood protons prior to their flowing into a region of interest to act as an endogenous “tracer.” (21)

Data from multiple studies suggest that mTBI results in a state of abnormal cerebral perfusion compared to healthy controls. The most frequent finding in studies utilizing ASL/pCASL across multiple age ranges (pediatric vs. adult), injury severities (mild/moderate/severe), timeframes (acute vs. chronic), and injury contexts is that of decreased perfusion. Wang et al. in 2016 demonstrated frontotemporal decreased CBF following subacute sport-related concussion in 18 young adult football players compared to 19 age-matched nonconcussed controls (23), as well as in a pediatric sample in 2015 (24). Clark et al. determined that reduced CBF was associated with decreased white matter integrity in 37 Veterans with chronic mild-moderate TBI (25). As severity of injury increases from mild to moderate and severe, there is a greater likelihood of decreased perfusion being present (26, 27). Newsome et al. found reduced CBF in right non-prefrontal regions in seven adolescents with chronic moderate-severe TBI, while Kim et al. in 2010 examined 27 chronic moderate-severe TBI patients and 22 matched controls with ASL and found globally decreased CBF in the TBI group, along with regional CBF reductions in posterior cingulate, thalamic, and frontal areas (26). This group also used resting and task-based ASL sequences to detect occipital and temporal hypoperfusion in 2012 in a cohort of 21 moderate-severe TBI patients (27). CBF remains abnormal into the chronic phase (28–32), and has been associated cognitive performance (27), symptom severity (32) and recovery (30).

However, several studies have found increased CBF in acute mTBI, especially in symptomatic cases. For instance, Doshi et al. in 2015 using ASL after acute mTBI found that in 14 patients with acute mTBI regional CBF was increased compared to 18 healthy controls (33). Similarly, Stephens et al. in 2018 found CBF increased in the left dorsal cingulate gyrus and left insula in 15 teenage athletes with subacute sport-related concussion compared with 15 age-matched controls (34). Finally, Barlow found that CBF was higher than controls in patients with symptomatic pediatric concussion, but lower than controls in asymptomatic pediatric concussion (30). It is apparent that a consistent pattern of change in CBF due to injury or recovery as measured by pCASL still needs to be established.

Cerebral perfusion and neurovascular coupling changes related to treatment may also be evaluated using pCASL sequences. Transcranial direct current stimulation (tDCS) (35), a type of noninvasive neuromodulation, has been shown in both animal and human studies to modulate CBF, depending on the parameters of the stimulation (excitatory vs. inhibitory, respectively) (36–40). Its promise as a treatment for cognitive deficits in mmTBI has been observed in multiple studies (41–44), and in a single small trial changes in perfusion tomography were seen following tDCS in a moderate-severe TBI population (45). However, no studies have measured cerebral perfusion with pCASL as a correlate of improvement with training or tDCS in mild or moderate injury (mmTBI). Therefore, this study aims to identify whether anodal tDCS applied to the left dorsolateral prefrontal cortex paired with a cognitive training protocol in mmTBI patients results in changes in CBF on pCASL sequences. It is hypothesized that anodal tDCS will result in regional perfusion increases, as well as improvements in cognitive performance and symptoms, compared to sham tDCS.

## Materials and Methods

Subjects with either mild or moderate TBI within the past 15 years were recruited via local brain injury clinics, brain injury advocacy centers, community flyers, and medical record search. Forty subjects aged 18–59 who had experienced mild or moderate TBI between 3 months and 15 years prior to study entry with persistent cognitive symptoms were screened and enrolled in the study. Subjects were randomized to receive either active or sham tDCS paired with cognitive training to improve executive functions and mood. Each patient underwent pre- and post-intervention testing, which included demographic assessment and medical history, TBI severity assessment, screening for contraindications to tDCS, postconcussive and behavioral symptom assessment, and neuropsychological testing. Of the forty subjects enrolled, a subset of twenty-four completed baseline and post-treatment magnetic resonance imaging scans, including pCASL. The UNM Health Science Center Institutional Review Board reviewed and approved this study.

### Inclusion Criteria

Subjects qualified for enrollment in the study if they met the following inclusion criteria: (1) age 18–59; (2) have suffered a mild or moderate TBI [“mild” defined as having had loss of consciousness (LOC) <30 min, received a Glasgow coma scale (GCS) score of between 13 and 15 upon ED evaluation (if available), and experienced <24 h of post-traumatic amnesia (PTA); moderate defined as LOC between 30 min and 24 h, GCS between 9 and 12, and PTA between 24 h and 7 days]; (3) were injured between 3 months and 15 years ago; (4) report at least 1 out of 4 cognitive symptoms on the Neurobehavioral Symptom Inventory (NSI). Potential participants were excluded from participation in this study for: (1) a history of other neurological disease, seizures, or psychosis; (2) history of recent (within 2 years) substance/alcohol dependence; (3) any discontinuity in skull electrical conductivity; (4) any implanted electrical device (e.g., pacemaker); (5) medical admission or hospital visit within the last 3 weeks; (6) change in any psychotropic medications in the previous 2 months; (7) inability to complete the protocol; (8) appointment of a legal representative, as assessed via direct inquiry of the subject or a designated trusted other; (10) inability to provide informed consent; (11) pregnancy, current incarceration, or limited English proficiency.

### Demographic Data

Basic demographic data regarding the subject were recorded, including age, sex, years of education, handedness, use of common stimulants such as caffeine, and brain injury severity. Subjects were asked to list any significant medical diagnoses, and any current medications, including psychotropics.

### Behavioral and Cognitive Battery

All neuropsychological testing was administered in the UNM Center for Brain Recovery and Repair Clinical Core by trained study personnel under direct supervision of clinical neuropsychologists. The pre- and post-intervention assessments consisted of the following tests: the Neurobehavioral Symptom Inventory (NSI) (3); the Hamilton Depression Rating Scale (HAM-D) (46); the Beck Depression Inventory-II (BDI) (47); the Posttraumatic Stress Disorder Checklist-Civilian version (PCL-C) (48); the Patient-Reported Outcomes Measurement Information System-29 (PROMIS) (49); the Glasgow Outcome Scale-Extended (GOS-E) (50); the Frontal Systems Behavior Scale (FrSBe) (51); Wechsler Adult Intelligence Scale-Fourth Edition (WAIS-IV): Digit Span and Coding subtests (52); the Test of Premorbid Functioning (TOPF) (53); the Hopkins Verbal Learning Test-Revised (HVLT-R) (54); and Test of Memory Malingering (TOMM) (55). These tests were selected due to their inclusion in the NINDS Common Data Elements for TBI, as well as their history of validation in TBI populations. The NIH Executive Abilities: Measures and Instruments for Neurobehavioral Evaluation and Research (EXAMINER) battery was utilized as a more specific assessment of executive functions (56), with subscores of fluency, cognitive control, and working memory, as well as an overall executive composite score. Testing was performed at study entry (Baseline Visit), immediately after completion of the intervention (Post-Treatment visit), and 1 month after study entry (Followup Visit). To mitigate fatigue, testing was performed over 2 days, and regular breaks were offered, with total time of testing ~5 h.

### Intervention

Participants were randomly assigned to either active or sham tDCS combined with executive function training tasks. A NeuroConn tDCS device (neuroCare Group GmbH, Munich, Germany) was used to administer tDCS. Sessions consisted of 30 min stimulation for 10 consecutive weekdays. The anodal electrode was placed on the left dorsolateral prefrontal cortex (DLPFC; F3 position, International 10–20 system) utilizing the Beam F3 targeting method (57) and the cathode was placed on the right upper arm just below the deltoid muscle to isolate anodal cerebral effects (58, 59). Neuroconn 5 cm^2^ rubber electrodes covered in 0.9% saline-soaked sponges were applied using elastic bandage. Current for the active condition was applied at 2.0 mA for a total delivered charge of 60 mA-min and a current density of 0.08 mA/cm^2^, consistent with guidelines describing acceptable safety and blinding at this current density (60). Active stimulation current was ramped up over 1 min at initiation, maintained for 30 min, and ramped down over 1 min at termination. Sham stimulation was delivered with an initial ramping up of current to 2.0 mA for 1 min, then ramping down and remaining at 0.02 mA for the duration of the session, to permit impedance monitoring. Double-blinding of subjects and study staff was accomplished using pre-determined stimulation codes entered into the stimulator. During tDCS application, subjects were assessed in terms of tingling, itching, mood, energy, pain, and wakefulness levels using visual analog 10-point scales. Sensation checks were performed every 10 min during the stimulation session.

All participants were administered a set of executive functions training tasks for 30 min during stimulation sessions. Each training session consisted of 10 min of the AX Continuous Performance Task (AX-CPT), a test of response inhibition, proactive and reactive cognitive control (61), and 20 min of a modified multimodal (visual/auditory) N-back working memory task (MMWM) (62), counterbalanced over the 10 sessions. These tasks were selected based on their relevance to the three executive functions comprising cognitive control (working memory, response inhibition, set shifting) (63, 64) and prior studies of cognitive control in TBI (65, 66).

### Cerebral Perfusion Imaging

MRI scans were performed during the baseline assessment visit, and on the day following completion of the stimulation protocol. MRI data was acquired on a 3T Siemens Trio scanner with a 32-channel head coil (see Supplementary Methods). High resolution T_1_-weighted (1 × 1 × 1 mm), T_2_-weighted (1.1 × 1.1 × 1.5 mm), susceptibility weighted images (1.00 × 1.00 × 1.50 mm) and fluid attenuated inversion recovery images (0.80 × 0.80 × 3.00 mm) were collected and reviewed by a blinded, board certified radiologist. Pseudo-Continuous Arterial Spin Labeling (pCASL; 45 tagged/untagged images) sequence was acquired (TR = 4,250 ms; TE = 11 ms; label offset = 90 mm; NEX = 1; slice thickness = 5 mm with 20% gap; bandwidth = 2,790 Hz/Px; labeling duration = 1,665 ms) with 20 interleaved slices for whole brain coverage (voxel size = 3.44 × 3.44 × 6.00 mm). A proton density (PD) sequence was also acquired to estimate T1 magnetization and scale CBF on a voxel-wise basis, with the post-labeling delay (PLD) and TR (5,200 ms) being the only parameters that varied across the pCASL (PLD = 1,800 ms) and PD scans (PLD = 3,400 ms).

To process pCASL for analysis, pCASL images were first despiked and registered to the pseudo-PD image using 2- and 3-dimension motion correction algorithms within the AFNI suite (67). Both images were then spatially blurred using a 6-mm Gaussian kernel. Each pre-processed labeled image was next subtracted from the paired control image, after which cerebral blood flow (CBF) was quantified using in-house software based on established parameters (blood/tissue water partition coefficient = 0.9 mL/g; longitudinal relaxation time of blood = 1,664 ms; labeling efficiency = 0.85; label duration = 1,665 ms) and algorithms (68). T1 magnetization correction and scaling of CBF was accomplished on a voxel-wise basis with the PD image. The quantified CBF data were then averaged and spatially transformed to standard stereotaxic space (69) using a non-linear transformation (AFNI 3dQwarp).

### Data Analysis

All data were double entered and underwent quality assurance checks prior to statistical analysis. Sample size was determined based on previously reported Cohen's *d* effect sizes of 1.2 for tDCS to induce improvements in cognition using a similar unicephalic electrode montage (70). Sample size calculation given this effect size indicated 13 subjects per group would achieve 80% power to detect a difference at the 0.05 level. A series of mixed-models repeated measures ANOVAs were utilized to analyze the pre- and post-intervention data, with between-subjects factors of GROUP (2 levels) and SEVERITY (2 levels), and a within-subjects factor of VISIT (3 levels). Main effects F values were calculated for each within-group and between-group factor as well as an interaction effect. Primary outcome variables for imaging were: (1) Global CBF value; (2) Regional CBF values in regions in the AAL Atlas (71) with interest around the anode. False Discovery Rate (FDR) corrections for multiple comparisons were performed within each hypothesis for the primary outcome variables. Correlations between change in regional CBF, cognitive, and symptom variables were calculated and examined for trends. All statistical analyses were run on R v3.5.3 (R Core Team, 2019) (72).

## Results

### Demographic Data

There were 10 subjects in the active tDCS group, and 14 subjects in the sham group, owing to the randomization protocol of the parent study. Baseline demographic variables, behavioral symptom scores, and neuropsychological test results are provided in Table 1. There were no significant baseline differences between the two groups.

**Table 1:** Baseline average demographic, behavioral, and neuropsychological performance characteristics of the active and sham groups.

	**Active (10)**	**Sham (14)**	**Sig (*p*)**
Male/female	6/4	9/5	0.84
Mild/moderate	8/2	10/4	0.64
Tobacco	9	13	0.82
Caffeine	5	9	0.51
Age	29.4	36.8	0.15
Education	14.7	14.7	0.99
Hand laterality	75	94.6	0.29
GOSE	6.5	6.2	0.51
TOMM	46.1	46.7	0.77
TOPF	107	105	0.82
BDI	15.2	17	0.64
HAM-D	15.3	16.5	0.73
NSI-somatic	9.3	9.1	0.96
NSI-cognitive	7.4	5.9	0.41
NSI-emotional	9.7	8.9	0.73
PCL-C	40.2	39.3	0.88
PROMIS-physical	18.1	16.1	0.21
PROMIS-anxiety	10.2	9.9	0.84
PROMIS-depression	9.2	8.6	0.72
PROMIS-fatigue	12.1	10.6	0.33
PROMIS-sleep	13.8	12.6	0.45
PROMIS-social satisfaction	11.1	12.1	0.56
PROMIS-pain interference	8.7	10.1	0.53
PROMIS-pain intensity	3.1	3.4	0.82
WAIS-DS	11	9.5	0.33
WAIS-CD	10	10.3	0.84
HVLT-recall	44.7	41.8	0.52
HVLT-delayed	38.4	45.1	0.27
HVLT-retention	38.3	48.7	0.11
HVLT-discrimination index	40.2	48.6	0.11
FRSBE-apathy	68.1	68.9	0.92
FRSBE-disinhibition	62.8	60	0.63
FRSBE-executive dysfunction	71.7	66.7	0.49
FRSBE-total	71.3	68.6	0.73
Examiner working memory	0.77	0.68	0.67
Examiner fluency	0.54	0.9	0.28
Examiner cognitive control	0.5	0.68	0.57
Examiner executive composite	0.65	0.95	0.28

### Neuropsychological Performance

**Figure 1:**
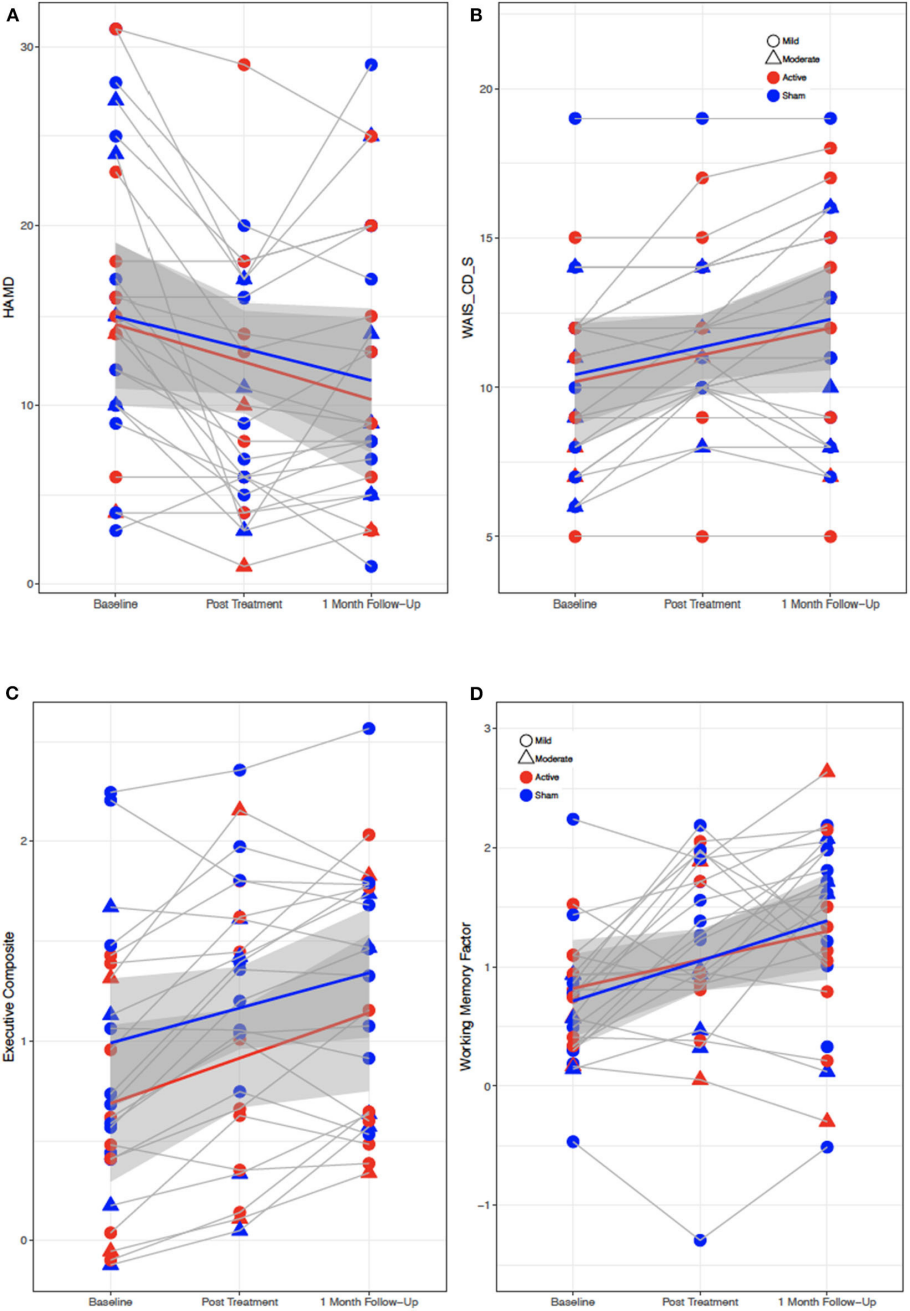
Behavioral and cognitive performance for all subjects from baseline to post-treatment to 1 month followup visit. **(A)** Depression symptoms (HAM-D). **(B)** Attention performance (WAIS-CD-S). **(C,D)** Executive function performance (EXAMINER Executive composite and working memory composite scores). Red, active; blue, sham; gray regions, standard error.

There was a significant main effect of VISIT observed in multiple behavioral and neuropsychological variables after correction for false discovery rate (FDR), including the BDI, HAM-D, NSI somatic and emotional subscores, PCL-C, WAIS-CD, and Examiner composite and executive scores (F = 7.0–18.9, all *p* < 0.01) (see Supplementary Table 1). Depression, anxiety, and postconcussive symptoms all decreased over time from Baseline to Post-Treatment Visit, while complex attention and executive functions improved. There were no main effects of GROUP nor interaction effects of GROUP × VISIT for any variables (see Figure 1).

### Global Perfusion

**Figure 2:**
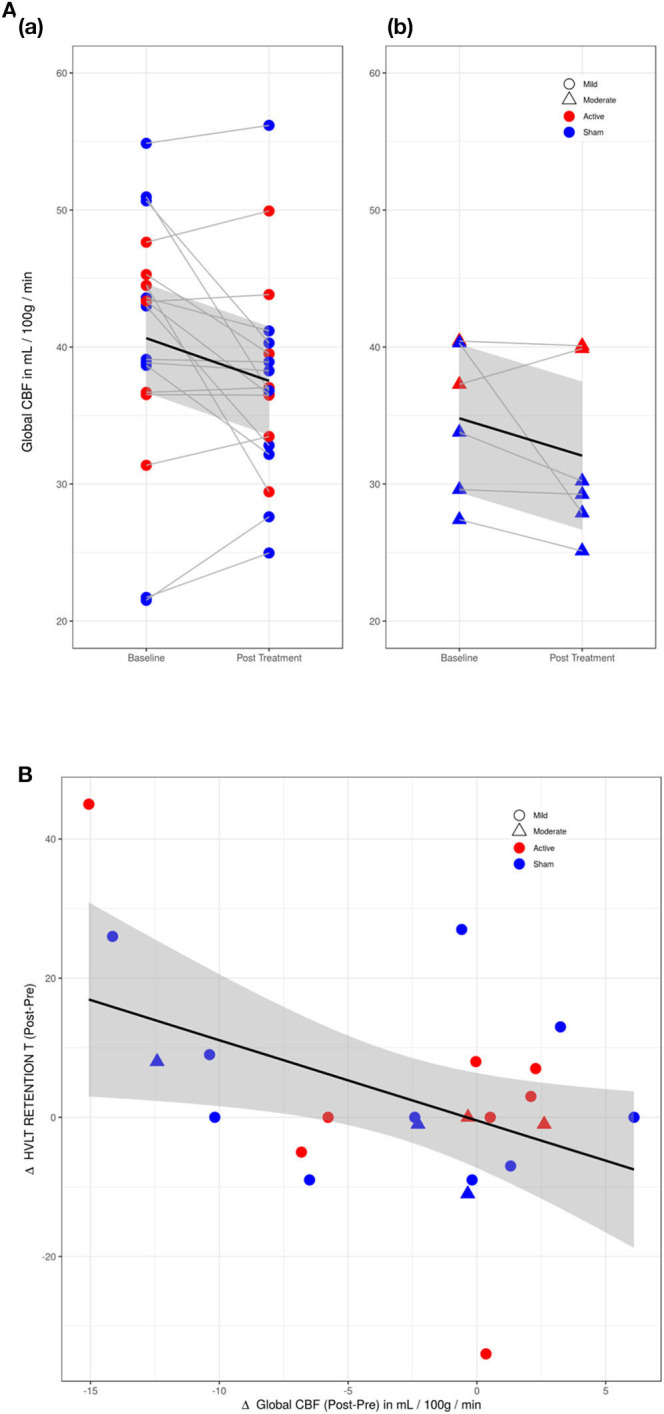
**(A)** Changes in global cerebral perfusion (CP) between baseline and post-treatment visits. (a) Mild TBI participants (circles). (b) Moderate TBI participants (triangles). Difference in CP between mild and moderate TBI participants was not significant. Red, active group; blue, sham group. **(B)** Scatter plot of Hopkins Verbal Learning Test (HVLT) retention score with cerebral perfusion (CP) for active (red) and sham (blue) groups. HVLT correlated mildly (*r* = −0.44) with reductions in CP.

Active and sham group mean values for global and regional CBF at Baseline and Post-Treatment time points are reported in Supplementary Table 1. A main effect of VISIT was observed, with a reduction of global CBF observed from Baseline to Post-Treatment Visit [F_(1, 23)_ = 6.417, p = 0.02] (see Figure 2A). While participants with moderate TBI had lower average perfusion values at both time points, the difference between subjects with mild vs. moderate TBI was not significant [F_(1, 21)_=2.42, *p* = 0.14] and VISIT^*^SEVERITY was not significant [F_(1, 22)_ = 0.02, *p* = 0.89]. Reduction in global CBF was weakly correlated with improvement on the HVLT Retention with *r* = −0.44, *p* = 0.03 (0.79) (FDR corrected p value in parentheses) (see Figure 2B).

### Regional Perfusion

**Figure 3:**
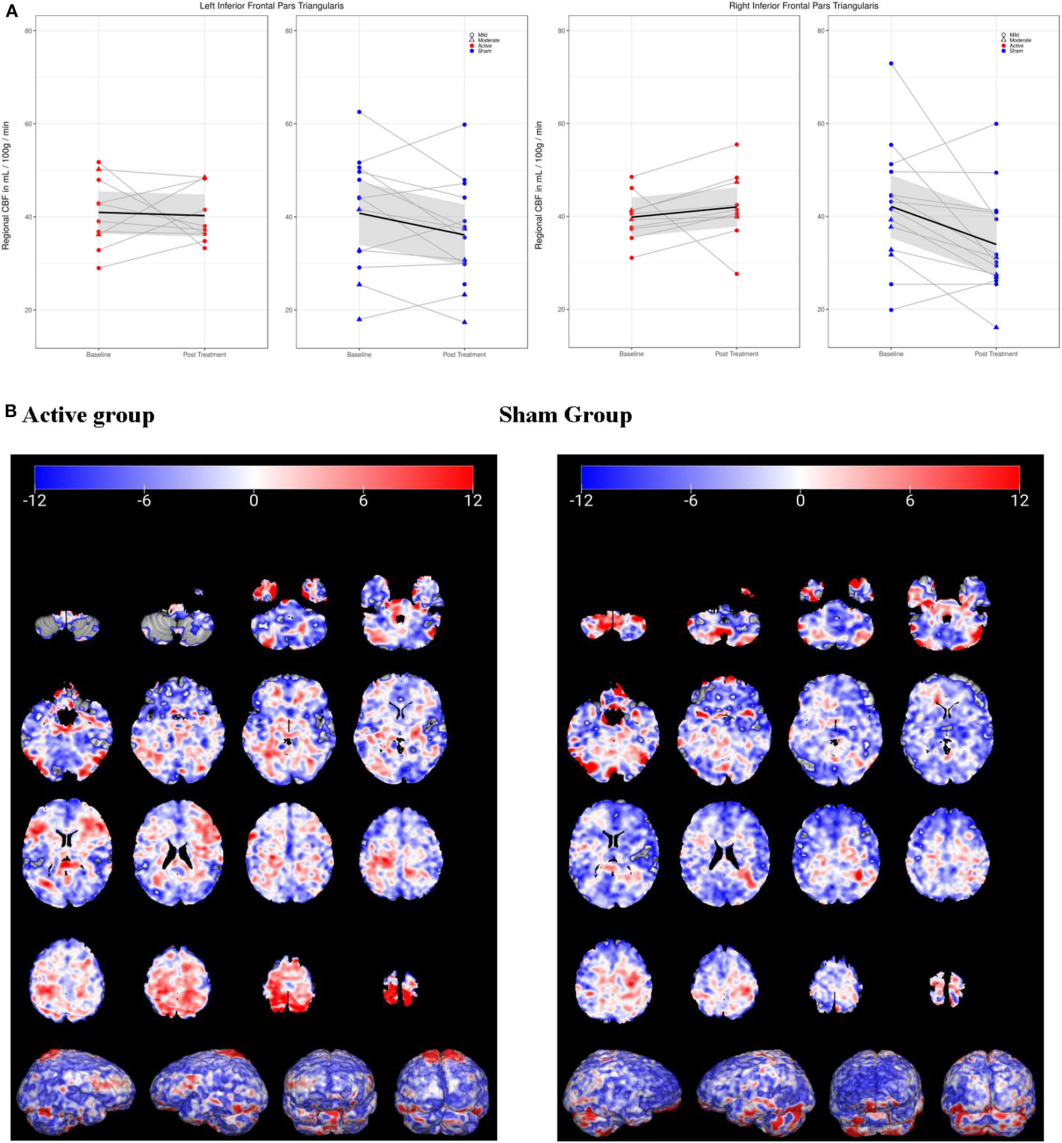
Cerebral perfusion (CP) in inferior frontal gyrus/pars triangularis (IFG) from baseline to post-treatment visit. **(A)** In left IFG, active group (red) demonstrated no change in CP, while sham (blue) decreased. In right IFG, CP increased in active group but decreased in sham group. **(B)** 3-D visualization of change in CP over time in active and sham groups. Blue, decreased CP; red, increased.

In the regional analysis of cerebral perfusion, active and sham groups demonstrated significantly different CBF changes in the inferior frontal gyrus (IFG) from Baseline to Post-Treatment Visits (see Figure 3). In the active group, perfusion remained static in the left IFG, and increased in the right IFG, while in the sham group, both left and right IFG demonstrated reductions in perfusion. Only in the right IFG was the difference between active and sham significant [F_(1, 22)_ = 6.12, *p* = 0.02(0.984)]. No regions passed FDR correction, and the only uncorrected regions with *p* < 0.05 were the cerebellum and the right IFG/pars triangularis.

To understand potential contributors to the observed CBF changes, an exploratory analysis was conducted of correlations between changes in right IFG CBF, neuropsychological test performance, TBI symptoms, and mood/anxiety/quality of life symptoms for both the active and sham groups (see Supplementary Figures 1a,b). In the sham group, mild to moderate correlations were observed between right IFG CBF and BDI/HAM-D (*r* = 0.19–0.45), NSI subscales (*r* = −0.31 to 0.52), PROMIS subscales (*r* = −0.04 to 0.48), WAIS-DS (*r* = −0.37), HVLT Retention scores (*r* = −0.37) and EXAMINER scores (*r* = −0.08 to −0.22). HAM-D was moderately correlated with performance on the HVLT Retention (*r* = −0.42), WAIS Digit Span (*r* = −0.43), and EXAMINER subscales (*r* = −0.25 to −0.55), while BDI tended to demonstrate correlations in the opposite direction.

In the active group, moderate to strong correlations were observed between right IFG CBF and NSI subscales (*r* = 0.46–0.85), BDI/HAM-D (*r* = 0.56–0.67), PROMIS subscales (*r* = −0.31 to 0.61), HVLT retention (*r* = −0.69), and FrSBe subscales (*r* = 0.42–0.75). Only mild and highly variable correlations were observed with the EXAMINER subscales (*r* = −0.45 to 0.3). A single outlier appeared to be driving most correlations, and excluding this data point reduced the strength of correlations to mild. Mild to strong correlations were found between depression scores (HAM-D, BDI) and NSI subscales (*r* = 0.27–0.78) and with performance on the HVLT Recall (*r* = −0.43 to −0.8) and Retention (*r* = −0.17 to −0.6).

## Discussion

In this small randomized sham study of patients with chronic mmTBI and cognitive deficits, cerebral perfusion changes were noted following a cognitive training paradigm and anodal tDCS to the left dorsolateral prefrontal cortex. Global CBF was noted to decrease over time, while increases in mood, attention, and executive function were observed, consistent with a hypothesis of increased cerebral efficiency. Baseline CBF values in our study were similar to other studies reporting decreased global CBF following injury. Our population of mild-moderate TBI patients manifested, on average, a global initial CBF rate of ~38–40 mL/100 g/min. This finding is consistent with that of previously cited studies, which found post-TBI regional CBF rates of 32–53 mL/100 g/min (26, 32, 33). However, changes in global CBF did not associate with objective cognitive performance or subjective mood measures, suggesting a more complicated relationship between clinical condition and generalized perfusion than initially hypothesized. There was no additional effect of tDCS seen, with both groups demonstrating decreases in global CBF regardless of receiving active or sham stimulation. This is not necessarily surprising, given that tDCS is applied in a targeted manner to a specific cortical region, and prior studies indicate that tDCS-induced changes in perfusion occur in specific regions rather than across the entire brain (37, 40).

In the regional analysis of cerebral perfusion, an effect of tDCS was observed, with reductions of CBF occurring in the right IFG in the sham group, and increases of CBF in the right IFG of the active group. Although FDR correction resulted in nonsignificance, this finding is still of some interest, as there is theoretical and empirical basis for altered right prefrontal and right fronto-parietal perfusion after mTBI (32, 73–75). The right frontoparietal network is implicated in lateralization of cognitive and emotional functions, including inhibition (76), visual attention, and emotional sham. Right frontal dysfunction has also been associated with several symptoms such as depression (77), anxiety (78), somatization (79), impulsivity (80), and distractibility (81), all of which may be seen in chronic mmTBI.

The effect of anodal tDCS on regional CBF in this study was consistent with other studies of excitatory tDCS and cerebral blood flow, in that an increase in perfusion was observed in the prefrontal cortices where current density is predicted to be highest (82). The larger CBF effect being observed in the right IFG as opposed to under the electrode on the left is somewhat paradoxical but may be explained by the strong functional connectivity typically observed between cortical regions and their homolog in the opposite hemisphere (e.g., interhemispheric transfer) (83, 84). While this regional finding is encouraging for tDCS having a potentially beneficial effect on perfusion and chronic symptoms of mmTBI, it did not correlate significantly with neuropsychological performance nor with subjective symptom report following treatment. There is a theoretical concern that if beneficial effects of cognitive training relate to reduced regional CBF, anodal tDCS may actually be counterproductive or deleterious to this process. However, the exploratory correlation analysis did not support this possibility, as correlation strengths between CBF and symptom/performance improvements in the sham and active group were approximately the same. Also of note in the exploratory analysis was that of the strongest correlations found were between emotion measures and executive function performance in both groups, pointing to a potential nonspecific mood benefit of the training or study protocol that may have obscured any contribution from the tDCS.

We consider our finding of decreased global and regional perfusion following the cognitive intervention somewhat paradoxical, considering that our study sample was already manifesting reduced CBF values at baseline. This raises the theoretical question of how decreased CBF can be both a marker of injury, as well as a marker of rehabilitation response. While it is possible that global CBF may have decreased over time independent of injury status or the intervention, CBF measured by pCASL has been shown to be relatively stable over time (85). Another possibility is that lower CBF is an adaptation to the injured state, and that with training, the adaptation is strengthened or amplified. Barlow et al. found that recovered patients after concussion manifested lower CBF values than controls, suggesting that recovery does not necessarily involve a return to original baseline CBF values (30). A second possibility is that while global CBF may be an accurate reflection of an injury condition, the recovery process may be occurring at a more regional level. This is suggested by our asymmetric CBF findings following tDCS and training: right frontal decreased perfusion, accompanied by left frontal increased perfusion, may be a recovery pattern for mood, cognition, and behavior, such as is seen in transcranial magnetic stimulation for depression (86, 87). A final consideration is that resting CBF measured by pCASL may fail to fully account for changes to dynamic cerebrovascular regulation and its relation to metabolic demand. Static reductions in cerebral perfusion after TBI may be accompanied by increases in CBF with effort on functional sequences such as task-based fMRI or cerebrovascular reactivity challenges (88, 89). It might be necessary to obtain measurements of both CBF and metabolic activity (i.e., positron emission tomography) in order to better understand how the brain is adapting to injury and responding to training (90). In summary, our findings suggest that the role of cerebral perfusion in the pathogenesis of and recovery from PPS continues to be complex.

This study was limited by its small sample size, uneven group numbers, heterogeneity of the clinical sample, and the lack of a non-treatment control group, limiting any conclusions that can be drawn. However, many patients with TBI fail to demonstrate any cognitive gains in rehabilitation, whereas this sample demonstrated robust improvements in multiple cognitive domains. Therefore, the prospect of a simple 10-day program of cognitive training on executive functions leading to objective improvements is cause for further study. Cerebral perfusion measured with pCASL represents a potential pathophysiologic target for rehabilitation paradigms in mmTBI such as cognitive training and tDCS.

## Data Availability Statement

The raw data supporting the conclusions of this article will be made available by the authors, without undue reservation.

## Ethics Statement

The studies involving human participants were reviewed and approved by UNM HSC HRRC. The patients/participants provided their written informed consent to participate in this study.

## Author Contributions

DQ was responsible for primary writing, literature review, and conceptualization of the study. JU, TJ, and AM were responsible for higher-level analysis of imaging and behavioral data. EB, JS-R, VF, RR, JW, and DG were responsible for data collection, curation, and primary analysis. MH, VC, RC, RY, CS, and JR provided input on study design, data collection methods, cognitive training, and stimulation protocols. All authors contributed to the article and approved the submitted version.

## Conflict of Interest

The authors declare that the research was conducted in the absence of any commercial or financial relationships that could be construed as a potential conflict of interest. The reviewer DB declared a past co-authorship with the authors AM, CS to the handling editor.
