# Supplementary material for: Cerebral Perfusion Effects of Cognitive Training and Transcranial Direct Current Stimulation in Mild-Moderate TBI
Source: Front Neurol. 2020 Oct 7;11:545174. doi: 10.3389/fneur.2020.545174 (PMC7575722; doi:10.3389/fneur.2020.545174)
Supplement: Supplementary file 1 [file Table_1.DOCX]

**Supplementary Table 1a. Repeated-measures ANOVAs of Behavioral and Neuropsychological variables.**

**(* = p < 0.01)**

| Measure | F_stat | df1 | df2 | p | p (FDR) |
| --- | --- | --- | --- | --- | --- |
| BDI | 7.465 | 2 | 46 | 0.002 | 0.007* |
| HAM_D | 18.941 | 2 | 46 | 0 | >0.001* |
| NSI Somatic | 7.027 | 2 | 46 | 0.002 | 0.009* |
| NSI Cognitive | 3.079 | 2 | 46 | 0.06 | 0.10 |
| NSI Emotional | 8.545 | 2 | 46 | 0.001 | 0.006* |
| PCL-C | 7.837 | 2 | 46 | 0.001 | 0.007* |
| PROMIS Physical Function | 3.071 | 2 | 46 | 0.06 | 0.10 |
| PROMIS Anxiety | 4.270 | 2 | 46 | 0.02 | 0.06 |
| PROMIS Depression | 3.369 | 2 | 46 | 0.04 | 0.09 |
| PROMIS Fatigue | 2.475 | 2 | 46 | 0.10 | 0.15 |
| PROMIS Sleep Disturbance | 3.981 | 2 | 46 | 0.02 | 0.06 |
| PROMIS Social Satisfaction | 2.404 | 2 | 46 | 0.10 | 0.15 |
| PROMIS Pain Interfernce | 4.827 | 2 | 46 | 0.01 | 0.04 |
| PROMIS Pain Intensity | 1.729 | 2 | 46 | 0.19 | 0.24 |
| WAIS Digit Span | 2.519 | 2 | 45 | 0.09 | 0.15 |
| WAIS Digit Symbol Coding | 14.338 | 2 | 45 | 0 | >0.001* |
| HVLT Recall | 1.001 | 2 | 45 | 0.38 | 0.45 |
| HVLT Delayed | 0.089 | 2 | 45 | 0.92 | 0.93 |
| HVLT Retention | 0.533 | 2 | 45 | 0.59 | 0.64 |
| HVLT Discrimination | 0.072 | 2 | 45 | 0.93 | 0.93 |
| FRSBE Apathy | 0.643 | 2 | 45 | 0.53 | 0.61 |
| FRSBE Disinhibition | 2.228 | 2 | 45 | 0.12 | 0.16 |
| FRSBE Executive Dysfunction | 3.977 | 2 | 45 | 0.03 | 0.06 |
| FRSBE Total | 2.237 | 2 | 45 | 0.12 | 0.16 |

| EXAMINER Exec Composite | 18.603 | 2 | 45 | 0 | >0.001* |
| --- | --- | --- | --- | --- | --- |
| EXAMINER Fluency Factor | 7.690 | 2 | 45 | 0.001 | 0.003* |
| EXAMINER Cog Control Factor | 2.984 | 2 | 45 | 0.06 | 0.09 |
| EXAMINER Working Mem Factor | 10.100 | 2 | 45 | 0 | 0.001* |

**Supplementary Table 1b. Global and regional cerebral blood flow values for active and sham groups at Baseline and Post-Treatment assessments. (Values in mL/100g/min; CBF = cerebral blood flow; IFG = inferior frontal gyrus; SD = standard deviation.)**

|  | Baseline Mean CBF (+/- SD) | Post-Treatment Mean CBF (+/- SD) |
| --- | --- | --- |
| Global |  |  |
| Active | 40.6 (5.1) | 38.6 (5.6) |
| Sham | 38.1 (10.4) | 34.4 (8.4) |
|  |  |  |
| Left IFG |  |  |
| Active | 41.0 (7.5) | 40.3 (6.0) |
| Sham | 38.7 (14.3) | 36.1 (11.2) |
|  |  |  |
| Right IFG |  |  |
| Active | 39.8 (5.0) | 42.0 (7.4) |
| Sham | 42.1 (13.2) | 34.0 (11.2) |
