# Supplementary figures and images for: Cerebral Perfusion Effects of Cognitive Training and Transcranial Direct Current Stimulation in Mild-Moderate TBI
Source: Front Neurol. 2020 Oct 7;11:545174. doi: 10.3389/fneur.2020.545174 (PMC7575722; doi:10.3389/fneur.2020.545174)

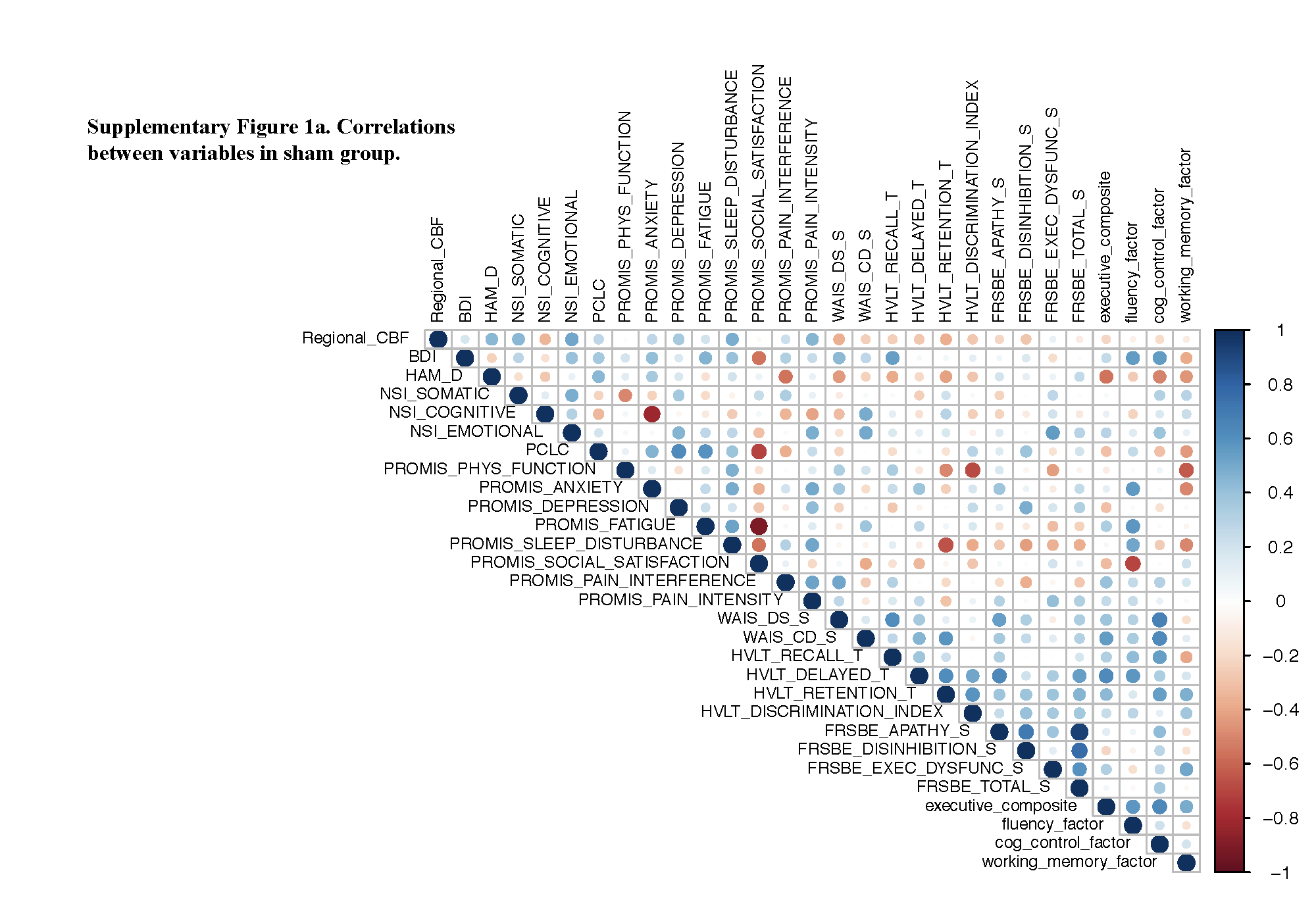

Supplement: Supplementary file 2 [file Image_1.TIFF]

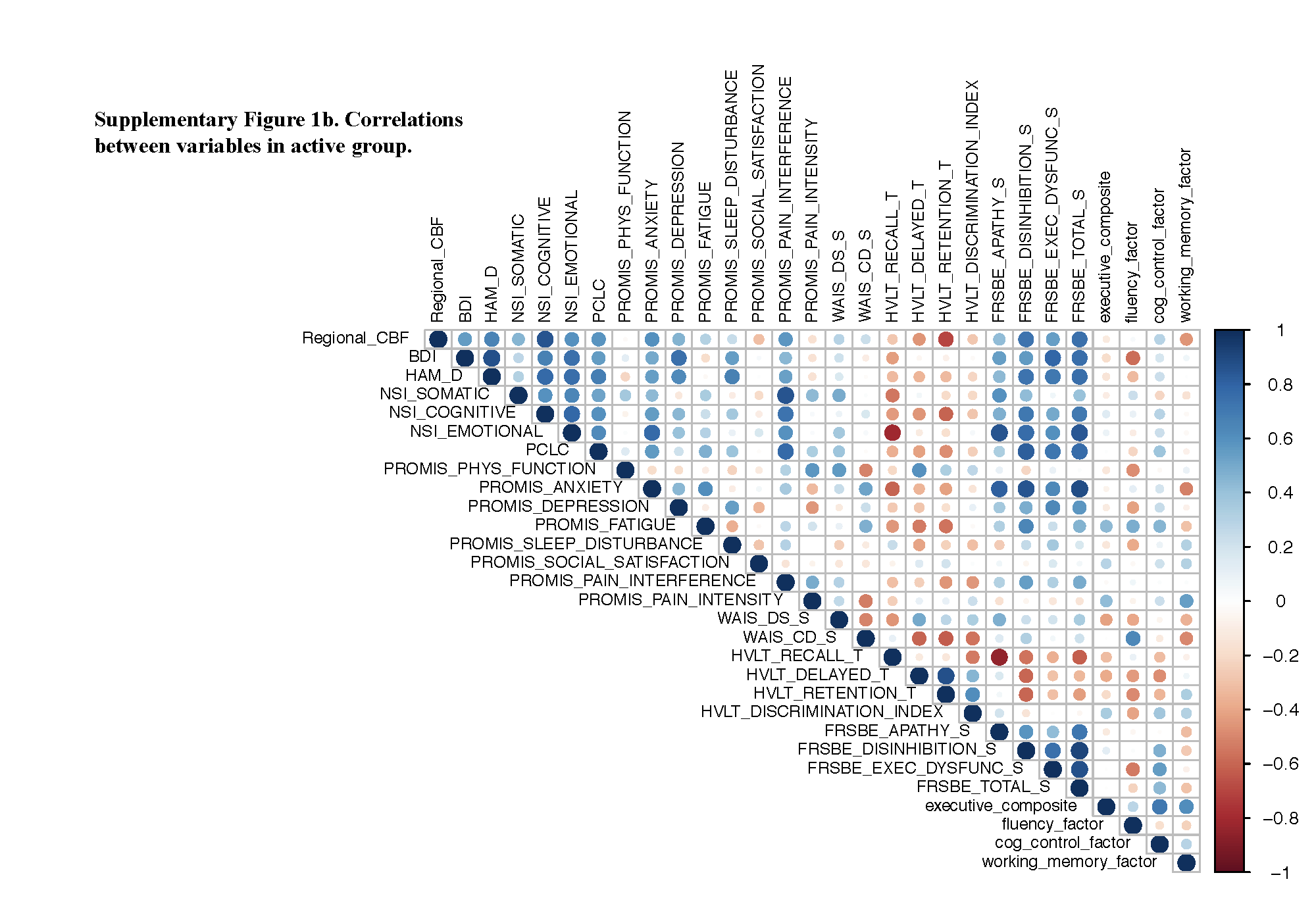

Supplement: Supplementary file 3 [file Image_2.TIFF]
